# Uses of Linguistic Context in Speech Listening: Does Acquired Hearing Loss Lead to Reduced Engagement of Prediction?

**DOI:** 10.1097/AUD.0000000000001515

**Published:** 2024-06-17

**Authors:** Leigh B. Fernandez, Martin J. Pickering, Graham Naylor, Lauren V. Hadley

**Affiliations:** 1 Department of Social Sciences, Psycholinguistics Group, University of Kaiserslautern-Landau, Kaiserslautern, Germany; 2 Department of Psychology, University of Edinburgh, Edinburgh, United Kingdom; 3 Hearing Sciences—Scottish Section, School of Medicine, University of Nottingham, Glasgow, United Kingdom.

**Keywords:** Context, Hearing loss, Prediction

## Abstract

Research investigating the complex interplay of cognitive mechanisms involved in speech listening for people with hearing loss has been gaining prominence. In particular, linguistic context allows the use of several cognitive mechanisms that are not well distinguished in hearing science, namely those relating to “postdiction”, “integration”, and “prediction”. We offer the perspective that an unacknowledged impact of hearing loss is the differential use of predictive mechanisms relative to age-matched individuals with normal hearing. As evidence, we first review how degraded auditory input leads to reduced prediction in people with normal hearing, then consider the literature exploring context use in people with acquired postlingual hearing loss. We argue that no research on hearing loss has directly assessed prediction. Because current interventions for hearing do not fully alleviate difficulty in conversation, and avoidance of spoken social interaction may be a mediator between hearing loss and cognitive decline, this perspective could lead to greater understanding of cognitive effects of hearing loss and provide insight regarding new targets for intervention.

## INTRODUCTION

Keeping up in conversations can be challenging for people with hearing loss, as prompt processing of speech is needed both to follow utterances between others, and to take part oneself. However, cues within the very structure of conversation can provide support. Critically, words are put into a “linguistic context”: a frame generated by semantics, syntax, and prosody (among others), which allows listeners to interpret words more efficiently than in isolation (Obleser 2014). Context can be particularly helpful for people with hearing loss because information that constrains the possible interpretation of an incoming signal allows the use of several cognitive mechanisms that can facilitate speech processing. However, we offer the perspective that hearing loss might hinder the use of one of these mechanisms, namely the use of context to make predictions of what is likely to come next.

We build this case by first distinguishing between three classes of cognitive mechanisms afforded by a linguistic context: postdiction, integration, and prediction. Second, we review context effects in challenging listening situations, focusing on both normal hearing listeners in degraded conditions, and listeners with hearing loss compared with age-matched controls, to identify the impact of these challenging listening situations on prediction. Third, we propose that reduced use of prediction is an unacknowledged source of difficulty for people with hearing loss, and consider three potential explanations: high cost, inefficiency, or use of alternative strategies. Last, we discuss how a nuanced distinction of postdiction, integration, and prediction fits into the Ease of Language Understanding model (ELU; Rönnberg 2003, 2022a), and propose potential avenues for further work.

We note that the claims we make in this perspective relate to use of prediction by adults with postlingual acquired hearing loss (i.e., occurring after language acquisition). However, due to a scarcity of prior research specifically addressing hearing loss, we draw from work across challenging listening situations. Particularly, we consider work on young normal hearing adults in clear versus degraded listening situations as well as work investigating older adults with and without postlingual hearing loss (using the terms “people with hearing loss” [PwHL] to contrast with “people with normal hearing” [PwNH]). While we acknowledge a likely interplay between aging and hearing loss on the use of context (Payne & Silcox 2019), we chose these contrasts to specifically draw conclusions about hearing loss without including age confounds.

## CONCEPT DEFINITION: POSTDICTION, INTEGRATION, AND PREDICTION

Our main interest in this article is distinguishing nonpredictive from predictive uses of context. Currently, the different cognitive mechanisms afforded by context are not well defined. Three relevant mechanisms discussed in prior work are postdiction, integration, and prediction (Ferreira & Chantavarin 2018; Pickering & Gambi 2018; Onnis et al. 2022; Rönnberg et al. 2022a).

Postdiction is the mechanism perhaps the most commonly discussed in hearing science. Postdiction can be used when a speech segment is missing or is not perceived as it occurs, and involves the listener retrospectively using context to activate linguistic information that they did not perceive in the input itself. Postdiction necessarily occurs after the misperceived target speech segment, and is a way to infer poorly heard speech after it has been presented. Postdiction can involve using subsequent speech (Gwilliams et al. 2018), earlier processed information, or both. For example, a listener might hear the sentence *My friend gave me an adorable …, that loves chasing squirrels* and retrospectively infer that the poorly heard two-syllable word with multiple plosives was puppy (either by reinterpreting the initial phrase, or using information later in the sentence).

Integration has been less extensively considered in hearing science, but has a long history in psycholinguistics (Ferreira & Chantavarin 2018). Integration is the incremental processing (of speech or written language) that allows a listener to perform bottom-up analysis on each new element as it arrives, and then combine it with what has come before. Importantly, integration is easier for words that are closely related to the prior context, but does not involve preactivation of those words (Onnis et al. 2022). Integration has been demonstrated by people processing final words more quickly in more constraining than less constraining sentences (Ehrlich & Rayner 1981; Schwanenflugel & Shoben 1985). We note here however that the distinction between integration and postdiction may relate to speed of processing, and there is debate regarding their independence (Onnis et al. 2022).

Last, prediction specifically and uniquely requires the preactivation of linguistic information (e.g., meaning, syntax, phonology, word form). In other words, listeners anticipate what is likely to occur in the near future without encountering (bottom-up) input that carries that information. For example, a listener might hear *The sun went behind a …* and predictively activate cloud. The advantage of making this prediction is that the word cloud is highly likely to occur next, and so the listener can “get ahead of the game” by reducing lexical competition and thus speed the processing of auditory input. Demonstrations of prediction require evidence of preactivation of a target (or potential referent) before it occurs (Altmann & Kamide 1999; Pickering & Gambi 2018). People can make predictions at different linguistic levels (Pickering & Gambi 2018; Huettig et al. 2022), and can incorporate prior knowledge, pragmatic information, and discourse-level information (McRae & Matuski 2009; Huettig et al. 2011). This perspective article specifically focuses on prediction of meaning (with paradigms often operationalizing this by constraining the upcoming word form).

While hearing science and psycholinguistics both have a rich literature investigating uses of context, inconsistent frameworks for interpreting findings make developing holistic theories difficult (as discussed in Payne & Silcox 2019). Specifically, hearing research typically distinguishes between postdiction and prediction (in the sense of whether information is retrospectively inferred, or not), whereas psycholinguistics typically distinguishes between prediction and integration (in terms of whether information is preactivated, or activated when encountered). We specify postdiction as occurring after hearing the target, integration as occurring when hearing the target, and prediction as occurring before hearing the target. Using consistent definitions across fields could both allow greater cross-fertilization and facilitate the development of more comprehensive theories of context use.

## USE OF CONTEXT IN CHALLENGING LISTENING SITUATIONS

We now examine the literature on context use in challenging listening situations, to identify whether any of this evidence can be definitively attributed to prediction as defined above. There is much research investigating behavior and cognition while listening in adverse conditions (Mattys et al. 2012), with two main categories of work being relevant for this perspective: that investigating the use of context by PwNH in degraded versus ideal listening conditions, and that investigating the benefit of context for PwHL versus age-matched PwNH. Of course, it is important to note that PwNH listening under degraded conditions is not a perfect proxy for PwHL, as a degraded listening situation changes the perceptual quality of the auditory stimuli without capturing other aspects of hearing loss such as the loss of spectral, temporal, and spatial resolution, and any other long-term cognitive changes brought on by hearing loss (Divenyi & Haupt 1997; Wayne & Johnsrude 2015). Nonetheless, drawing from both bodies of work sheds broader light on the topic.

### Normal Hearing in Degraded Conditions

The effect of context on speech processing in degraded listening situations has been researched in various ways. One approach involves presenting speech in noise and measuring the intelligibility of different sorts of sentences. In such work, sentences provide either a high or a low level of context, and the benefit of that context has been robustly demonstrated through greater accuracy on the final word in the high than low context condition (Kalikow et al. 1977; Pichora-Fuller 2009; Obleser & Kotz 2010). Another approach is the phonemic restoration paradigm, which makes use of the illusion that, based on context, listeners report hearing parts of words that were removed from the auditory signal (Warren 1970). Most of this work uses speech in quiet, finding listeners to be more likely to report utterances as intact in a constraining linguistic context (Miller & Licklider 1950; Samuel 1981), though restoration has also been found in mild-moderate levels of degradation (i.e., down to 16-channel vocoding; Başkent 2012; Benard & Başkent 2014, 2015). But while this work demonstrates the benefit of context on intelligibility in degraded conditions, it does not demonstrate that the effect must be due to prediction.

Other studies use the visual world paradigm (VWP), an eye-tracking paradigm that provides insight into real-time linguistic processing and can be used to test prediction. In the VWP, participants view an array of images (or a scene) while listening to an utterance. One or more of the images relate to the utterance. The time course of looks to the images is analyzed to identify at which point people look more toward the target image, and discard the others. Specifically, if people look at the target (more than other images) before it is referred to, this would provide evidence for prediction; if after, this could be due to either integration or postdiction. Wagner et al. (2016) investigated the role of a semantically constraining verb on phonological competitors and semantic competitors in both natural speech and degraded speech using a noise-band-vocoder. While they do not provide enough information about timing to demonstrate whether prediction occurs, they found that participants used context both to integrate semantic information earlier and to rule out phonological competitors earlier in the normal listening condition than the degraded listening condition. Hence the use of context to support disambiguation between both semantic and phonological competitors was impaired when the speech was degraded.

Furthermore, Failes and Sommers (2022) used the VWP to explore how predictions were updated when the target of a sentence was degraded. Clear sentences were presented with only the final target word in noise, enabling optimal opportunity to correctly process context, but a degraded target. Both younger and older PwNH used contextual information and looked at the appropriate image at a similar time before the target word was spoken (e.g., looked toward the box while hearing “*She puts the toy in the*…”). But when contextual information led to a prediction that was not confirmed (e.g., the sentence ended “*She puts the toy in the fox*” ), older PwNH were more reluctant to revise their initial interpretations (i.e., gaze lingering on the wrong object), suggesting that in this population poor auditory input may lead to over-reliance on their predictions. Such an effect could be explained by the “noisy-channel” hypothesis (Levy 2008; Gibson et al. 2013), which holds that listeners operate with a level of uncertainty, and use sentence-level and background information to rationally infer intended meaning. Older adults with more linguistic experience, and experience resolving uncertainty, may therefore show greater reliance on prediction than younger adults (Baltaretu & Chambers 2018; Cutter et al. 2022).

Together, these findings suggest that in ideal listening situations, PwNH are able to make predictions in real time, but in degraded listening situations their use of contextual information is impaired. Use of prediction may also be impacted by individual abilities such as working memory capacity assessed via verbal and spatial working memory tasks (Huettig & Janse 2016; Nitsan et al. 2019; Nitsan et al. 2022). Thus, while predictions may be helpful, and even over-relied on when available (Failes & Sommers 2022), generating predictions in degraded listening situations may not be feasible (although other benefits of context may be retained).

### Hearing Loss

Given that PwHL are constantly faced with suboptimal listening situations, it is likely that effects of degradation and load extend to this population. While a range of work purports to discuss prediction and hearing loss, most or all of this in fact investigates the benefit of a constraining (or “predictable”) linguistic context, rather than specifically assessing the use of prediction. Thus, such work may indicate the effects of hearing loss on integration or postdiction, rather than prediction. We suggest that there is no published research explicitly investigating prediction during listening in PwHL.

In research into the intelligibility of speech in noise (that assesses performance after the stimuli have been heard), PwHL have been found to benefit substantially from linguistic context (Pichora-Fuller et al. 1995). Furthermore, though PwHL typically perform worse in intelligibility tests than PwNH, this difference is reduced for highly constraining stimuli, suggesting that the context benefit is even greater for PwHL than for age-matched PwNH controls (Pichora-Fuller et al. 1995; Gordon-Salant & Fitzgibbons 1997; Benichov et al. 2012). But while most studies compare only the extremes of low and high contextually constraining stimuli, it is possible that hearing loss also affects how much contextual information is necessary to elicit a context benefit. A nuanced study by Benichov et al. (2012) addressed this issue by investigating recognition thresholds of masked target words in low-constraint stimuli (e.g., “The cigar burned a hole in the FLOOR”) medium-constraint stimuli (e.g., “The boys helped Jane wax her FLOOR”), and high constraint stimuli (e.g., “Some of the ashes dropped on the FLOOR”) (Benichov et al. 2012). In the low constraint condition, PwNH did best, participants with mild hearing loss fell in the middle, and participants with moderate hearing loss did worst. In the medium-constraint condition, PwNH or mild loss performed at ceiling, and in the high-constraint condition, participants with moderate hearing loss also reached ceiling. Thus, low and middle-level constraints were not as beneficial for participants with moderate hearing loss as for PwNH, but stronger constraints benefited both groups. As the measurements took place on the target word, none of the effects can definitively be attributed to prediction.

Gating studies (Grosjean 1980), which present a sentence up to the start of its final word, and then present the final word in increasingly longer segments until it is identified, show that hearing loss leads to slower identification, particularly in low-context sentences (Wingfield et al. 1991; Perry & Wingfield 1994; Stine & Wingfield 1994; Lash et al. 2013; Moradi et al. 2014; Molis et al. 2015). This occurs even when participants wear hearing aids (Moradi et al. 2014). Furthermore, phonemic restoration studies found that although people with mild hearing loss were able to restore altered speech in a similar manner to those with normal hearing, people with moderate hearing loss showed no such restoration (Başkent et al. 2010). Again, these results could just as well be due to integration (or postdiction) as prediction.

While we assert that to date, no work has directly addressed prediction as defined earlier in PwHL,* a range of evidence is nonetheless compatible with reduced use of prediction with hearing loss. A first hint that PwHL may not be using prediction in the same way as PwNH comes from studies of the mechanisms by which listeners generate predictions. One proposed mechanism of generating predictions is via the language production system (Pickering & Garrod 2013), whereby listeners simulate the processes required to produce the heard speech and run a forward model to identify what is likely to come next. This “prediction-by-production” theory builds on the growing body of work indicating motor involvement in speech listening (Wilson et al. 2004; Möttönen & Watkins 2009), as well as work suggesting that this involvement provides a means of coordinating social actions with others (Novembre et al. 2014; Hadley et al. 2015). Strikingly, in a transcranial magnetic stimulation study, Panouillères and Möttönen (2018) found that while older adults with good hearing showed motor-engagement of the tongue motor area while listening to speech, this was not the case for the older adults with poorer hearing. A behavioral study using an articulatory suppression paradigm also suggested reduced motor-engagement in PwHL, consistent with reduced prediction by production (Slade et al. 2022). Further indication of an altered balance of top-down (contextual information) and bottom-up (acoustic information) processing in PwHL comes from the neural speech tracking literature, which has consistently shown enhanced tracking of the acoustic envelope of heard speech in PwHL (Decruy et al. 2020). PwNH show enhanced neural tracking for low contextually constraining compared with high contextually constraining speech (Molinaro et al. 2021), presumably due to greater reliance on bottom-up than predictive processing. In the same way, the enhanced neural tracking in PwHL overall could indicate greater reliance on bottom-up processing and reduced engagement of prediction. Thus, there are indications from two different neural mechanisms related to predictive processing that prediction use may be atypical in hearing loss.

## PROPOSITION THAT HEARING LOSS LEADS TO REDUCED PREDICTION

On the basis of the evidence reviewed above, we suggest that reduced use of prediction contributes to the difficulty that PwHL experience keeping up in conversation. None of the research investigating context use in PwHL demonstrates prediction, and there are in fact several indications that the mechanisms involved in prediction are less active in PwHL. Furthermore, when PwNH listen to speech in conditions that are somewhat comparable to those experienced by PwHL (e.g., when input is degraded, or cognitive resources decreased), they show reduced use of context during real-time speech processing (Huettig & Janse 2016; Nitsan et al. 2022). Hence while PwHL would potentially have most to gain from predictive, top-down, language mechanisms (Kuperberg & Jaeger 2016), it is unclear that they are able to make use of them.

A recent account argues that there are two stages to prediction (Pickering & Gambi 2018), with the first being a relatively resource-free and automatic means of making associative predictions, and the second being a more resource-demanding and non-automatic means of tailoring those predictions to the situation. We suggest that for PwHL, reduced audibility would affect first-stage predictions, but that even in cases when stimuli were fully audible, second-stage predictions would be impaired due to competition between real-time language processing and resource-intensive predictive processing including tailoring based on external nonlinguistic information. Indeed, second-stage prediction appears to be slower in other cognitively demanding listening situations such as in second-language speakers relative to first-language speakers (Corps et al. 2023). As prediction requires the use of potentially resource-demanding mechanisms in a quick and time-bounded manner, PwHL may show reduced prediction while nonetheless benefiting from context at later stages, hence the reported context effects in studies that measure performance after target words (see Use of context in challenging listening situations).

We suggest that there are three main reasons that the use of prediction may be reduced in PwHL, which we take in turn. These explanations are not mutually exclusive. First, the “high cost” explanation proposes that in PwHL, the resources required to make predictions exceed the available resources. In PwNH, degraded input and increased cognitive load delay integration and reduce prediction (Huettig & Janse 2016; Wagner et al. 2016), and these effects may be similar PwHL. Furthermore, it has been found that the increased cost of processing degraded auditory stimuli impacts listeners’ abilities to store and maintain information about previously processed words (Rabbitt 1968, 1991; Piquado et al. 2010). Poorer encoding of heard speech could further compound listeners’ difficulty generating predictions. Finally, because PwHL take more time to process speech (Wendt et al. 2015), the time that is available to generate predictions is reduced, meaning that predictions would need to be generated more quickly to remain “ahead of the game,” or over a longer time scale (i.e., to a point further ahead, increasing the chance of inaccuracy). Predictions in PwHL may therefore be too slow, or, to be quick enough to be helpful, require even more resources than for PwNH.

Second, the “reduced reliability” explanation proposes that prediction accuracy is lower for PwHL and thus engagement of prediction may be reduced. It has previously been suggested that use of prediction is dependent on its utility (Kuperberg & Jaeger 2016). Hearing loss leads to degraded auditory input, and thus reduces the reliability of the context that is perceived. When listening to a sentence, mishearing the first few words can lead to inappropriate constraints being applied to upcoming input, and thus inaccurate predictions (Marrufo-Pérez et al. 2019). Hearing loss may therefore substantially reduce the benefit of engaging prediction, which, over time, may alter the value of generating predictions for PwHL.

Third, the “alternative strategy” explanation proposes that reduced prediction is a natural consequence of greater focus on other speech processing mechanisms. It could, for example, be that postdiction is engaged more often and more strongly for PwHL than PwNH, and thus prediction is down-weighted as a priority. The exact reason for the down-weighting is not entirely clear, but could be a consequence of cost or unreliability. Alternatively, it may be that processing of other communicative cues such as speech rhythm, facial expression, or body movement is prioritized if they are particularly useful for PwHL (i.e., high benefit with low cost). Thus, resources may be diverted away from prediction during speech listening and toward the processing of other cues, but for reasons relating to the benefit of the alternative strategies rather than anything intrinsic to prediction itself.

## FUTURE DIRECTIONS: RELATION TO THE EASE OF LANGUAGE UNDERSTANDING (ELU) MODEL AND FURTHER WORK

### The ELU Model

Clearer distinctions between different uses of linguistic context could lead to substantial progress in our understanding of language processing in hearing loss, and allow us to identify specific sub-processes ripe for intervention in individual interlocutors. Currently in the hearing field, the ELU model is the best-known and most comprehensive model of speech processing in hearing loss and degraded conditions (Rönnberg 2003; Rönnberg et al. 2013, 2019). Importantly, the ELU model acknowledges the facilitatory use of linguistic context (though focusing on benefit occurring when speech is degraded), and we suggest that considering this model in relation to the specific mechanisms discussed earlier would drive further insight into the cognitive impacts of hearing loss.

To summarize, the ELU model provides an explanation for how single words are identified through reference to memory, postdictive processes, and predictive processes. It proposes that if a word is heard perfectly it will automatically resonate with a representation in semantic long-term memory (via a buffer named RAMBPHO), and that this “match” enables understanding. However, if a word is degraded, or there is a mismatch between the input and the representation in semantic long-term memory for some other reason (e.g., due to an unfamiliar signal processing algorithm in a hearing aid), postdiction is called upon through an explicit “slow and deliberate” processing loop (Rönnberg et al. 2019, p. 249). In the model, postdiction uses working memory processes to combine what was heard with subsequent clarifications, enabling reconstruction of meaning from the degraded input. Prediction, on the other hand, is described as a “fast and implicit” process (Rönnberg et al. 2019, p. 249) that involves pre-tuning the input buffer for likely words (that is, preactivating those words). Unfortunately, much of the evidence referenced in the ELU literature as relating to prediction is equally compatible with an explanation in terms of postdiction or integration, exemplifying the ubiquitous conflation of prediction and integration in hearing science.

While the definitions from the ELU are consistent with those provided in this article, the original ELU figures depicted prediction as a part of the postdictive repair process rather than a separate mechanism (Rönnberg et al. 2019, p 248). However, the most recent version of the ELU model denotes prediction as a separate mechanism (driven by comprehension of prior linguistic information) that is applied at an earlier point of language processing (). To enable preactivation, prediction feeds into speech processing before comparison between input and memory, and we suggest that this prediction could push the representation of words in memory closer to the threshold necessary for auditory input to match that word during the comparison stage. While integration mechanisms are not explicitly featured in the new ELU model, they could fit into the framework at the match/mismatch stage by allowing contextually related words to more easily pass the matching threshold. By specifying such distinctions and using consistent definitions of the cognitive mechanisms afforded by linguistic context across disciplines, we can ask new questions and potentially reveal new ways to support listening ease.

**Fig. 1.**
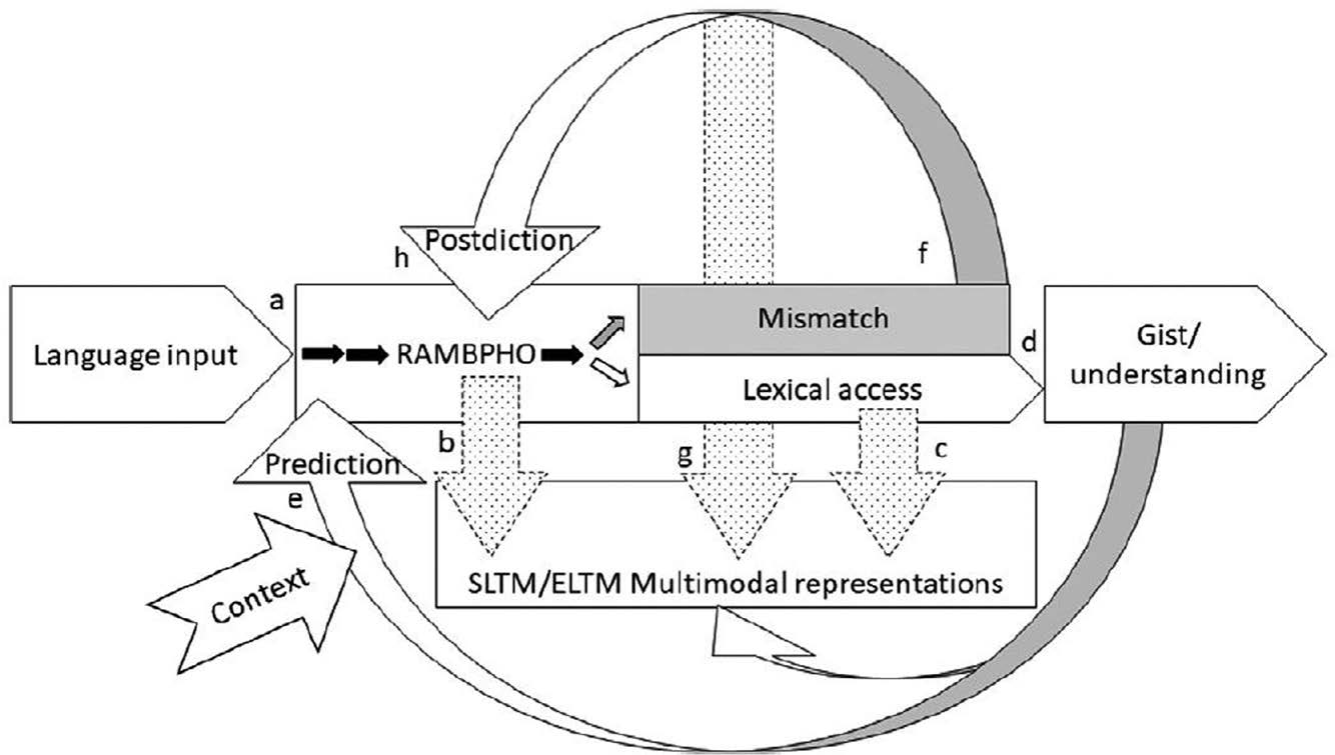
The Ease of Language Understanding Model (from Rönnberg et al. 2022a, p 200).

### Discussion and Further Work

We acknowledge that the majority of research investigating the use of context in speech listening involves stimuli and tasks that are highly artificial, which could lead to concerns about the generalizability of these findings and our claims of impaired prediction contributing to difficulty in conversation (Keidser et al. 2020; Beechey 2022). For example, predictable targets are often located at the end of standalone sentences, which are not representative of most everyday language use. Yet effects have been validated in more naturalistic speech listening conditions, with VWP findings replicating in complex virtual reality formats and when target sentences are embedded in longer and more varied monologues (Heyselaar et al. 2021). Because prediction must be assessed before a word is heard, unnatural paradigms are unfortunately likely to continue, but we emphasize the importance of combining such work with assessment in more typical conversational conditions to ensure that findings are relevant to everyday language use.

We propose that reduced use of prediction is consistent with a variety of behaviors reported in conversation for PwHL. As prediction has been proposed to underlie the rapid turn-taking that occurs in conversation (Levinson 2016), reduced use of prediction could be implicated in the more variable turn timing of PwHL in comparison to people with PwNH (Sørensen et al. 2019; Petersen et al. 2022). Reduced use of prediction could also underlie a reduced ability to follow conversational switches (Whitley et al., manuscript in preparation, Reference Note 1), because recognizing that a turn is about to end is important to allow a listener to prepare for the next turn (Holler & Kendrick 2015; Hadley & Culling 2022). If such difficulties are indeed due to reduced use of prediction, this is a cognitive impact of hearing loss that has been underexplored and consequently underexploited as a target for intervention.

It is worth considering whether there would be a cost of making incorrect predictions for people with hearing loss. While language models have shown an accuracy of approximately 30% when estimating the next word in a corpus (Cevoli et al. 2022), meaning that many words are indeed predictable, the cost of incorrect predictions remains an open question. If prediction errors are costly, then directing resources to prediction could be a poor strategy. Currently, there is little consensus on prediction costs even in PwNH (Delong et al. 2012; Delong et al. 2014; Frisson et al. 2017; Chow & Chen 2020). We hope that this perspective serves as an impetus to better understand how these mechanisms work more generally, as well as how they may be altered as a result of hearing loss and its cognitive impacts.

Ultimately, if prediction is reduced with hearing loss, this is an intriguing avenue for intervention. Training of predictive abilities in PwHL could provide a means of alleviating some of the speech-listening difficulties associated with hearing loss, impacting both conversation success and quality of life. For several decades, audiologic training has included the use of context (Pichora-Fuller & Levitt 2012), and only small adjustments to such training would be needed to increase focus on prediction. In the same light, training the use of context in other more flexible ways could also be useful, for example training PwHL to use contextual information postdictively when beneficial. Further research examining how to best develop these skills, or alter the weighting of top-down over bottom-up processing, could therefore provide the basis for a novel rehabilitation approach that could be rolled out with immediate effect. In addition, hearing technology could also be adjusted to support prediction, for example by algorithmically emphasizing specific prediction-enhancing cues (e.g., regularizing speech rhythm, or increasing prosodic range, to enhance turn-end cues). Further work investigating whether and how prediction mechanisms could be developed and supported in PwHL would therefore not only provide new insights for researchers, but could also benefit clinical hearing care.

In sum, in this article we have presented a distinction of three different ways that linguistic context can be used to benefit speech processing: (1) to enable postdiction (i.e., inferring words that were not fully heard after they occurred), (2) to facilitate integration (i.e., combining words with what came before at the point they occur), and (3) to generate predictions (i.e., preactivating targets before they occur). We have also presented evidence that these mechanisms may work differently (or be emphasized to a greater or lesser degree) in the presence of hearing loss. Last, we discussed how these distinctions fit into the ELU model, and have suggested future work that could lead to novel audiologic training and hearing technology development.

## ACKNOWLEDGMENTS

The authors thank Jerker Rönnberg for a constructive conversation on the Ease of Language Understanding Model which shaped parts of the discussion.

## Notes

* In one VWP study, children with prelingual hearing loss and cochlear implants and/or hearing aids made similar predictions to age-matched children with normal hearing (Holt et al. 2021). But as prelingual hearing loss affects language acquisition (Kasai et al. 2012), this behavior may be quite different from that of adults with postlingually acquired hearing loss.
